# *Zygochrimnushenryi*, a new genus and species from South America (Hemiptera, Lygaeoidea, Lygaeidae)

**DOI:** 10.3897/zookeys.796.20702

**Published:** 2018-11-15

**Authors:** Harry Brailovsky

**Affiliations:** 1 Departamento de Zoología, Instituto de Biología, Universidad Nacional Autónoma de México, Apdo. Postal No. 70153, Ciudad de México. Universidad Nacional Autónoma de México México Mexico

**Keywords:** Insecta, Heteroptera, Lygaeinae, Neotropics, new genus, new species

## Abstract

*Zygochrimnushenryi*, new genus and new species, is described from Ecuador and Peru, and included in the lygaeid subfamily Lygaeinae. Dorsal and lateral view photographs of *Melanopleurus*, *Melanopleuroides*, and *Zygochrimnus* are provided to allow comparison of the three closely related genera; photographs of the posterior view of the pygophore and the paramere of the new species are included.

## Introduction

The lygaeid subfamily Lygaeinae is large, with a worldwide distribution; its members are often aposematically colored with contrasting red and black or orange and black. Species of the subfamily are characterized by having the hemelytra impunctate; the membrane of forewing usually with a distinct basal cell; the hindwing with hamus and subcostal vein; all abdominal spiracles dorsal; and sutures of the abdominal venter straight or nearly so, and all reaching lateral margin of abdomen (Slater 1985, Henry 1997, Baranowski and Slater 2005, Henry et al. 2015).

The Western Hemisphere members of this subfamily were revised, twenty-two genera were recognized and their phylogenetic relationships discussed by A. Slater (1992). Since then only one new genus has been added, *Melanopleuroides* A. Slater & Baranowski, 2001, from Dominican Republic (Slater and Baranowski 2001).

In this paper, one new genus and one new species from Ecuador and Peru are described, illustrated, and compared with the related genera *Melanopleurus* Stål, 1874 and *Melanopleuroides* A. Slater & Baranowski, 2001.

This manuscript recognizes Dr. Thomas J. Henry’s brilliant scientific career. I met him for the first time in 1976 and since then, our academic bond has strengthened and a true friendship has developed. We have collected together in different localities throughout Mexico for many years and shared our passion for insects. I have seen him grow and attain an outstanding position within the global field of heteropteran systematics.

## Materials and methods

The following abbreviations are used for the institutions cited here: University of California, Riverside, California, USA (**UCR**); Instituto de Biología, Universidad Nacional Autónoma de México (**UNAM**). The holotype is deposited in UCR; paratypes are in UNAM. The classification and terminology proposed by Slater (1992) are followed.

Pictures were taken with a Nikon D200 camera.

## Taxonomy

### Zygochrimnus gen. n.

Taxon classificationAnimaliaHemipteraLygaeoidea

http://zoobank.org/2F652C97-2092-4B0E-913A-07F1DE1CC2A4

Figure 1B, E, G

#### Type species.

*Zygochrimnushenryi* sp. n.

#### Diagnosis.

Distinguished among New World Lygaeinae by dorsal surface of body densely clothed with long, stout, upright hairs; body subovoid, robust; head dorsally black with yellow discoidal spot at vertex; and pro-, meso-, and metapleuron densely punctate.

#### Description.

**Figure 1.**
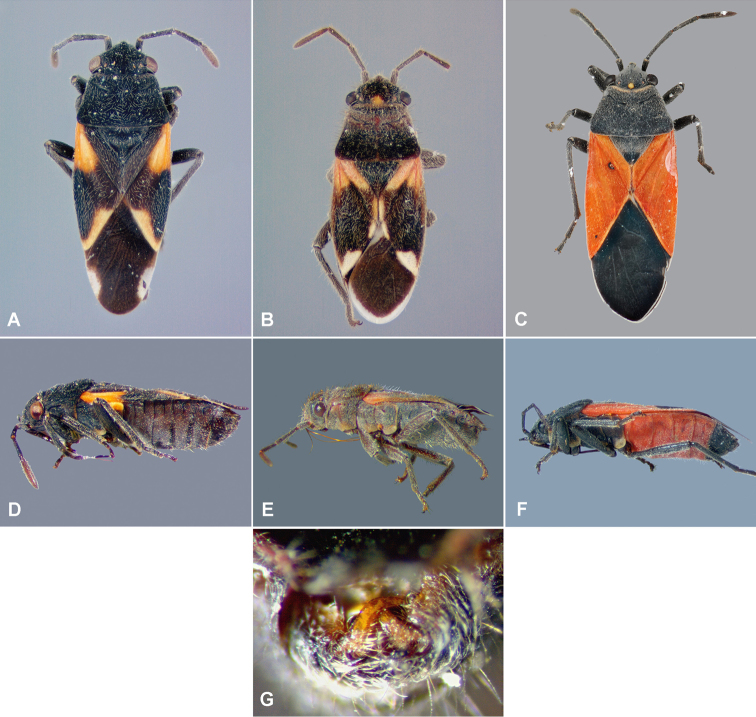
*Melanopleuroidesdominicanus* A. Slater & Baranowski female (**A** dorsal side **D** lateral side); *Zygochrimnishenryi* gen. & sp. n. male (**B** dorsal side **E** lateral side **G** genital capsule and paramere); *Melanopleurusbicolor* Herrich-Schäffer female (**C** dorsal side **F** lateral side).

**Male.** Moderately robust, subovoid, widest across middle of abdomen, medium sized, length less than 5 mm. ***Head.*** Sloping downward, wider than long, vertex convex; ocelli much closer to eyes than to each other; ocellus small, slightly raised above surface; eyes hemispheric, not protruding, with posterior margin touching frontal angles of pronotum; buccula moderately produced; rostrum reaching posterior margin of metasternum; rostral segment I thickest, touching anterior border of prosternum; segment II slightly thinner, III and IV thinner than II and about equally thick. ***Thorax.****Pronotum*. Trapezoidal, wider than long; anterior border slightly concave; frontal angles gently raised, touching posterior border of eyes; posterior margin straight, with shallow but distinct depression laterally; anterolateral margins slightly sinuate; callus indistinct, defined primarily by punctate areas immediately before and behind; callar impressions unbranched, obliquely sinuate, angled toward frontal pronotal angles; median carina obsolete. *Scutellum*. Wider than long; median carina T-shaped with stem clearly exposed and arms barely defined; lateral fovea deep. *Thoracic pleura*. Propleuron divided into three parts by dorsoventral impressions, anterior and posterior parts coarsely punctate, median part impunctate; mesopleuron divided into anterior and posterior punctate parts by shallow impunctate area; metapleuron with anterior half impunctate, posterior half coarsely punctate, posterior border almost straight, and posterolateral angle produced, somewhat rounded; ostiolar peritreme well developed. *Legs*. Unarmed; femora elongate. *Hemelytron*. Slightly surpassing apex of abdomen; costal margin barely emarginate; veins slightly raised. ***Abdomen.*** Anterolateral scars absent. *Genitalia*. Genital capsule subcircular in cross section. Paramere with well-developed caudolateral lobe; blade broad; posterior projection conical (Figure 1G).

*Surface and Vestiture*. Impunctate except for punctures immediately before and behind callus and on pro-, meso- and metapleuron. Head dorsally, pronotum, scutellum, clavus, corium, and legs densely clothed with long, stout, golden, upright hairs; head ventrally, thorax and abdomen densely clothed with short, fine, decumbent, silvery hairs.

#### Female.

Unknown.

#### Discussion.

This new genus keys to *Melanopleurus* and *Melanopleuroides* in A. Slater (1992) and Henry et al. (2015). The general appearance is similar, with eyes not on stalks; callus not depressed; hemelytral membrane without hyaline apical area; pronotum without four transverse depressions behind calli; scutellum not swollen; pronotal disk convex, finely punctate and almost entirely black to brownish. *Melanopleuroides* can be distinguished by having the head in dorsal view entirely black without yellowish discoidal spot at vertex; the discoidal spot is clearly defined in the other two genera (Fig. 1A, D). In *Zygochrimnus* gen. n., the head dorsally, pronotum, scutellum, clavus and corium are densely clothed with long, stout, golden, upright hairs; clavus, corium and abdominal sterna III-V are black with or without yellow to orange marks; and pro- meso-, and metapleuron are densely punctate (Fig. 1B, E); in *Melanopleurus* the head dorsally, pronotum, scutellum, clavus and corium are clothed with short, fine, decumbent hairs; the clavus, corium and abdominal sternite III-V are entirely bright orange to reddish orange; and the pro-, meso- and metapleuron are impunctate (propleuron sometimes with a few scattered punctures) (Fig. 1C, F).

### Zygochrimnus henryi sp. n.

Taxon classificationAnimaliaHemipteraLygaeoidea

http://zoobank.org/EE6DEB77-E12B-4DEB-80F7-B51F0D826C87

Figure 1B, E, G

#### Material.

***Type material.* Holotype, male.** PERU: Amazonas, 1 km W Siempre Viva, 5°44'40"S, 78°40'26"W, 475 m, 14 Dec 2005, F. Andrews (UCR). **Paratypes**: PERU: Cajamarca, 5 km N, Tambopata, 5°25'40"S, 78°48'8"W, 560 m, 15 Dec 2005, F. Andrews 1 male (UNAM). ECUADOR: Manabi, Rio Chico, Puerto Lopez, 5 m, 29 Mar 2002, I. G. Tapia, 1 male (UNAM).

#### Diagnosis.

As in generic diagnosis.

#### Holotype description.

***Dorsal coloration.****Head*: Dark reddish brown to black with yellow discoidal spot at vertex; antennal segments I-III pale reddish brown, IV dark orange. *Pronotum*: Anterior lobe dull black with wide dull orange stripe behind anterior border; posterior lobe shiny reddish brown with wide dull orange irregular stripe behind posterior margin of anterior lobe. *Scutellum*: Black with apex (stem) shiny orange. *Hemelytra*: Clavus with basal half, claval margin bordering scutellum and claval commissure pale yellowish-white; posterior half with pale reddish-brown triangular spot; claval margin bordering scutellum pale yellowish white, contrasting with pale reddish brown margin bordering corium; claval vein concolorous with surrounding area; costal margin of corium pale yellowish white basally and apically, and pale reddish brown between; apical margin of corium pale yellowish white interrupted at middle by pale reddish-brown short stripe; outer spot broader than inner one; hemelytral membrane pale brown with basal angle and outer margin white to yellowish white. *Abdomen*: Connexivum and dorsal abdominal segments dark brown to dark reddish brown.

*Ventral coloration*. *Head*: Black; buccula black with inner margin dark orange; rostral segments pale castaneous orange. *Thorax*: Propleuron black with anterior and posterior margin and acetabula dark orange; mesopleuron and metapleuron black with posterior margin and acetabula dark orange; pro-, meso- and metasternum dark brown; ostiolar peritreme pale yellowish white. Coxae black with dark reddish-orange reflections; trochanter dark reddish orange; femora dark reddish orange with apical third darker; tibiae dark reddish orange; tarsi pale castaneous orange. *Abdomen*: Abdominal sterna and genital capsule dark reddish brown.

#### Paratype variation.

1, pronotal disk entirely reddish brown. 2, basal third of clavus and corium pale yellowish orange. 3, acetabula dark yellow. 4, mesopleuron black. 5, metapleuron black with outer margin dark yellow.

#### Measurements.

**Male holotype.** Body length 4.70 mm; head length 0.55 mm; width across eyes 1.25 mm; interocular distance 0.80 mm; interocellar distance 0.52 mm; preocular distance 0.52 mm; antennal segments: I, 0.32 mm, II, 0.77 mm, III, 0.67 mm, IV, 0.85 mm; pronotum: length 1.07 mm; width across humeral angles 1.32 mm; scutellum: length 0.52 mm; width 0.82 mm; maximum width across abdomen 1.80 mm.

#### Etymology.

Named after Thomas J. Henry, indefatigable and remarkable collector, specialist in several groups of Heteroptera and editor of an entomological journal. Dr. Henry is a great human being, excellent friend, and a true model to be followed by the future generation of entomologists and systematists.

#### Distribution.

Known from Ecuador and Peru.

## Supplementary Material

XML Treatment for Zygochrimnus

XML Treatment for Zygochrimnus henryi
